# Integrative application of licorice root extract and melatonin improves faba bean growth and production in Cd-contaminated saline soil

**DOI:** 10.1186/s12870-024-05954-0

**Published:** 2025-01-08

**Authors:** Shimaa A. Abd El Mageed, Ali A. S. Sayed, Ahmed Shaaban, Khaulood A. Hemida, Abdelsattar Abdelkhalik, Wael M. Semida, Ibrahim A. A. Mohamed, Mohammed A. H. Gyushi, Yasmine H. Abd Elmohsen, Taia A. Abd El Mageed

**Affiliations:** 1 Agronomy Department, Faculty of Agriculture, Fayoum University, Fayoum, 63514 Egypt; 2 Botany Department, Faculty of Agriculture, Fayoum University, Fayoum, 63514 Egypt; 3 Botany Department, Faculty of Science, Fayoum University, Fayoum, 63514 Egypt; 4 Horticulture Department, Faculty of Agriculture, Fayoum University, Fayoum, 63514 Egypt; 5 Vegetable Research Department, Agricultural and Biological Institute, National Research Center, Dokki, Giza 12622 Egypt; 6 Soil and Water Department, Faculty of Agriculture, Fayoum University, Fayoum, 63514 Egypt

**Keywords:** Salinity stress, *Vicia faba*, Natural biostimulant, Melatonin, Anatomical structure, Cadmium contamination, Antioxidant enzymes

## Abstract

**Background:**

Globally, salinity poses a threat to crop productivity by hindering plant growth and development via osmotic stress and ionic cytotoxicity. Plant extracts have lately been employed as exogenous adjuvants to improve endogenous plant defense mechanisms when grown under various environmental stresses, such as salinity. This study investigated the potential of melatonin (Mt; 0, 50, and 100 mM) as an antioxidant and licorice root extract (LRE; 0.0 and 3%) as an organic biostimulant applied sequentially as a foliar spray on faba bean (*Vicia faba* L.) grown in cadmium (Cd)-contaminated saline soil conditions [Cd = 4.71 (mg kg^− 1^ soil) and ECe = 7.84 (dS m^− 1^)]. Plants not receive any treatment and sprayed with H_2_O were considered controls. The experimental treatments were laid out in strip plot in a randomized complete block design replicated thrice, where the LRE and Mt were considered as vertical and horizontal strips, respectively. Growth characteristics, photosynthetic pigments, nutrient uptake, physiology and metabolic responses, anatomical features, and yield were assessed.

**Results:**

Cadmium (Cd) and salinity-induced stress significantly altered leaf integrity, photosynthetic efficiency, total soluble sugars (TSS), free proline (FPro), total phenolic, DPPH, and total soluble proteins (TSP), non-enzymatic and enzymatic antioxidants, growth characteristics and yield-related traits. However, the application of LRE + Mt considerably improved these negative effects, with higher improvements were observed due to application of LRE + Mt_100_. Application of LRE + Mt significantly reduced hydrogen peroxide (H_2_O_2_) accumulation, lipid peroxidation and Cd content in leaves and seeds, all of which had increased due to Cd stress. Application of LRE + Mt significantly mitigated the Cd-induced oxidative damage by increasing the activity of reactive oxygen species (ROS) scavenging enzymes such as superoxide dismutase, catalase, ascorbate peroxidase, and glutathione reductase, in parallel with enhanced ascorbate and reducing glutathione content. Exogenous application of LRE + Mt significantly increased osmolyte content, including FPro, TSS, and total phenols and mitigated Cd-induced reduction to considerable levels.

**Conclusions:**

Our findings showed that LRE + Mt increased *V. faba* plants’ morphological, physiological, and biochemical properties, reducing Cd stress toxicity, and promoting sustainable agricultural practices.

**Clinical trial number:**

Not applicable.

**Supplementary Information:**

The online version contains supplementary material available at 10.1186/s12870-024-05954-0.

## Background

Faba bean (*Vicia faba* L.) is a critical pulse crop grown in the Mediterranean region as a rotational crop [1]. It is a valuable source of protein, energy, minerals, and fiber for human consumption and livestock feeding [2]. It also plays an important role as a rotation and mixed crop in enhancing soil fertility through the symbiotic fixation of atmospheric nitrogen (N), which is critical in low-nitrogen environments and supports sustainable production of cereal grains and intercrops with vegetables and sugarcane [3]. It is grown worldwide in a variety of cropping systems including dry grain (pulse), green grains/pods, and a green-manure legume [4]. Faba bean seeds are widely consumed for human food in many developing countries including China, Turkey, Egypt, Ethiopia, and Central America [5]. It ranked fifth with 5.4 Mt from 2.6 Mha, following pea, chickpea, common bean, and lentil [4, 6]. Faba bean is the fourth most frequently cultivated pulse crop in Egypt [6], and its production is greatly affected by environmental stresses such as saline stress [7].

Soil salinity is the most destructive abiotic factor lead to reducing cultivated land, crop yield, and quality worldwide [8]. Of all the world’s irrigated land, 19.5% is salt-affected, and 11% has become saline, posing a sever threat to sustainable agriculture and food security [9, 10]. Salt-affected areas are anticipated to expand by 50% by 2050 as a result of saline water irrigation, rising sea levels, and the potential of global warming [11, 12].

Salinity is also a significant constraint on faba bean production [7]. It impacts plant performance through osmotic stress, which causes ionic imbalance by accumulating large concentrations of Na^+^ and Cl^−^ in the cell, resulting in ion toxicity [13]. Osmotic effects from high Na^+^ and Cl^−^ influx can lead to Ca^2+^ or K^+^ deficiency, oxidative stress, membrane injury, degradation of leaf chlorophyll, induces stomatal closure, reducing leaf photosynthetic capacity and eventually yield [14]. Plants adapt several mechanisms to reduce osmotic and ionic effects, including Na^+^ exclusion from shoots [15], Na^+^ and Cl^−^ compartmentation in shoot and roots cells to reduce ion toxicity in chloroplast and delay senescence [16], and osmotic adjustment through the accumulation of organic and inorganic solutes in the cytoplasm to detoxify reactive oxygen species (ROS) for membrane stability, and retaining turgor to support plant growth [15].

As one of the most hazardous contaminants, cadmium (Cd) ranks seventh among the most dangerous poisonous metals [17], posing serious hazards to human, animal, and plant health [18, 19]. Both anthropogenic activities (metal electroplating industries, cement industries, batteries, power stations, mining, paints, combustion emissions, sewage sludge, smelters, superphosphate fertilizers, and pesticides), and natural causes (sewage, weathering of cadmium-containing rocks, volcanic eruptions, and forest fires) release Cd to the environment [20]. Cd can be easily taken in by roots, subsequently transported to above-ground parts and infiltrating the food chain, potentially leading to substantial risks to human health [21].

Exposure to Cd leads to a range of detrimental effects on plants, including physiochemical, morphological, and structural alterations including inhibition of lateral root formation, stomatal density, and chlorosis [22]. It generates osmotic stress in the plant by decreasing stomatal conductance, transpiration, and relative water content [23]. In addition, the toxicity of Cd has adverse impacts on plants by hindering CO_2_ fixation, and decreasing the synthesis of chlorophyll and photosynthesis [3]. It also disrupts the absorption and transport of nutrients, leading to decreased yield [21, 24]. Cd toxicity stimulates excess production of reactive oxygen species (ROS), which damage cell membranes and disrupts organelles and biomolecules [25]. Furthermore, Cd interferes with the uptake and transport of Mn, K, P, Ca, and Mg [24, 26]. These physiological disruptions induced by Cd collectively result in impaired growth and ultimately decrease crop yield [20, 23].

Severe heavy metals (HMs) and salinity stress impede plant growth by producing high levels of ROS, including non-radicals like hydrogen peroxide (H_2_O_2_), and free radicals like superoxide (O_2_^•−^), and hydroxyl (OH^−^) [24, 27, 28]. Excessive ROS levels under oxidative stress destroy chlorophyll, lipids, proteins, and DNA, resulting in cell damage and death [27]. However, plants employ enzymatic antioxidants [e.g., glutathione reductase (GR), catalase (CAT), superoxide dismutase (SOD), and peroxidase (POD)] and non-enzymatic antioxidants [e.g., ascorbate (AsA), proline, glutathione (GSH), carotenoids, and α-tocopherol (α-TOC)], to combat oxidative stress-induced damage [29, 30]. Previous studies on dual stresses of HMs and salinity have been reported [31]. In these reports, antioxidant defenses cannot maintain plant growth under severe stress [32]. Thus, exogenous application is required to enhance plant stress tolerance.

Melatonin (Mt; N-acetyl-5-methoxytryptamine) is an indoleamine identified in plants in 1995, and recognized to have a multitude of regulatory roles in humans, animals, and plants [33]. Melatonin is an ecofriendly biomolecule that can enter cell compartments due to its small size and a high solubility in water and lipids. The application of Mt is regarded as an alternative and cost-effective strategy for improving plant tolerance to abiotic stressors such as salinity, pH, and heavy metals [34]. Mt is a potent antioxidant against reactive oxygen and nitrogen species (ROS/RNS). Furthermore, Mt acts as a protective agent against abiotic and/or biotic stressors [33, 35, 36]. Although each stressful agent induce specific physiological responses, Mt, in general, enhances physiological processes such as plant growth, rooting, seed germination, photosynthesis, osmoregulation, anti-senescence, primary and secondary metabolism, and plant hormone regulation [33]. Moreover, it causes multiple alterations in gene expression.

Exogenous applications of plant natural extracts such as licorice root extracts [37] have been utilized for pre-sowing seed treatment and plant foliar spray to improve performance in saline conditions. Licorice (*Glycyrrhiza glabra*) root extract (LRE) is an organic biostimulant rich in secondary metabolites, antioxidants, phytohormones, and minerals that influence physiological, biochemical, and molecular mechanisms to promote plant adaptability, growth, and yield under salinity [37]. LRE contains antioxidants, osmoprotectants, sugars, vitamins, mineral nutrients, amino acids, selenium, and phytohormones (Table S1). Because of its high glycyrrhizin content (potassium [K] and calcium [Ca] salts of trihydroxy acid and glycyrrhizic acid), LRE can be employed to improve plant tolerance to abiotic stresses such as salinity stress [37]. It effectively reduces ROS production, and neutralizes oxidative stress. It also can improve growth, productivity, anatomy features, nutrient contents, and the levels enzymatic and non-enzymatic antioxidants, reduce lipid peroxidation, increase membrane integrity, and plant production [37, 38].

Excess Cd accumulation and salinity stress conditions can be toxic to the growth of *V. faba*, resulting in a significant reduction in yield. Therefore, it was hypothesized that the application of Mt and LRE could mitigate these negative effects. In this context, this study investigates the potential role of LRE and Mt applied sequentially as a foliar spray on growth, photosynthetic pigments, nutrient uptake, physiology and metabolic responses, anatomical features, and yield of *V. faba* grown in Cd-contaminated saline soil conditions.

## Materials and methods

### Site description, experimental climate conditions, and soil characterization

Two field experiments were conducted for two winter successive growing seasons (2022/23 and 2023/24), at a private field (latitudes 29°06’ and 29°35’ N, longitudes 30°26’ and 31°05’ E.), in Fayoum province of Egypt. Table 1 shows the physical and chemical properties of the soil used in this study, which were analyzed according to the methods of Klute and Dirksen and Page et al., [39, 40]. The soil analysis indicates that it is sandy loam with a slightly alkaline (pH = 7.71) soil and low organic matter content (OM = 0.89%). The soil electrical conductivity (ECe) is 7.84 dS m^− 1^, and the Cd content is 4.71 mg kg^− 1^, being Cd-contaminated saline soil. The available nutrients were; 56.21, 4.52 and 46.46 (mg kg^− 1^ soil) for N, P and K, respectively.

**Table 1:** Initial physicochemical properties of the soil used in this study

Soil physical characteristics^1^					
Depth (cm)	Particle size distribution			Texture	*ρ*_*b*_ (g cm^− 3^)
	Sand (%)	Silt (%)	Clay (%)		
0–30	73.33	12.15	14.52	Loamy sand	1.54
*Soil chemical properties*					
pH		7.71	Available nutrients		
ECe (dS m^− 1^)		7.84	Cd (mg kg^− 1^ soil)		4.71
CEC (cmole kg^− 1^)		11.92	N (mg kg^− 1^ soil)		56.21
CaCO_3_ (%)		4.12	P (mg 100 g^− 1^ soil)		4.52
OM (%)		0.89	K (mg 100 g^− 1^ soil)		46.47

^**1**^*ρ*_*b*_, Bulk density; ECe, Electrical conductivity; CEC, Cation exchange capacity; OM, Organic matter

### Field experimental design, treatment details, and agronomic management

The current experiment consists of two studied factors, namely exogenous foliar spraying of licorice root extract (LRE) and melatonin (Mt) to *V. faba* plants grown in Cd-contaminated saline soil. Thus, the on-field experiment comprises six (2 LRE levels × 3 Mt concentrations) experimental treatments replicated thrice and laid out in strip plot arrangements in a randomized complete block design (RCBD). The LRE levels and Mt concentrations were allocated to the vertical and horizontal strips of the experimental design, respectively. The Mt was applied at three concentrations, i.e., 0 Mm (Mt_0_; only distilled water), 50 mM (Mt_50_), and 100 mM (Mt_100_) three times day-15 intervals beginning from 20 days after sowing (DFS) to run-off. Moreover, the LRE was foliarly applied (+ LRE; 1%) three times in day-15 intervals beginning from 21 DFS to run-off or not applied (-LRE). The foliar spraying times concur with the BBCH 13/15 (3rd 5th leaves unfolded), BBCH 23/24 (3–4 side shoots detectable), and BBCH 32/33 (2–3 visibly extended internodes) phenological stages based on the BBCH, a German abbreviation for Biologische Bundesanstalt, Bundessortenamt und CHemische Industrie-codification keys of *V. faba* [41]. The commercial licorice root was kindly obtained from Sekem Company, Cairo, Egypt, while the Mt was purchased from Sigma-Aldrich (Seelze, Germany), and dissolved in ethanol to prepare the solutions. The extraction of licorice root active ingredients was performed as outlined by Desoky et al. [42]. A weight of 100 g of licorice root was air-dried and soaked in 2 L of distilled water for 1 d at 50 °C, then filtered using Whatman filter papers no. 42, and the final volume of 10 L was reached by distilled water. The LRE’s chemical constituents are shown in Table S1. For LRE and Mt, tween-20 (0.1%, *v/v*) as a nonionic surfactant agent was compiled with the spray solution to ensure optimal penetration into leaf tissues.

The *V. faba* L. (cv. Sakha 1) seeds used in this study, were obtained from the Egyptian Ministry of Agriculture and Land Reclamation. The seeds were sterilized in sodium hypochlorite solution (1%; v/v) after that the seeds were washed in distilled water and left in air temperature to dry. The seeds were sown on November 5 and 10 for both winter growing seasons and harvested on April 1, 2023, and April 6, 2024, respectively. The *V. faba* plants were fertilized with mineral nitrogen (N), phosphorus (P), and potassium (K) by applying 48 kg N ha^− 1^, 72 kg P_2_O_5_ ha^− 1^, and 58 kg K_2_O ha^− 1^ in the form of ammonium nitrate (33.5% N), calcium superphosphate (12% P_2_O_5_), and potassium sulfate (48% K_2_O), respectively, during both growing seasons. The full superphosphate amount was incorporated into the soil before sowing, the ammonium nitrate was applied in two equal bates at 28 and 40 DFS, while the full amount of potassium sulfate was added with the first ammonium nitrate bate. Each experimental plot area was 18 m^2^ including three sowing rows (10 m length × 0.6 m width for each) and equally spaced apart by 0.2 m between rows. The *V. faba* seeds were sown 3–5 cm aside from the drip line at about 5 cm depth, drip-irrigated with drip lines (Ø= 1.6 cm) with an inner dripper distance of 0.25 m and a flow rate of 3.2 L h^− 1^ at a working pressure of 0.15 MPa. Two irrigation dropper lines were positioned above each sowing row in which each dropper corresponded to two *V. faba* plants, achieving a planting density of 333.33 thousand plants ha^− 1^. All agricultural practices, except for our experimental treatments, were performed according to the recommendations of the Egyptian Ministry of Agriculture and Land Reclamation.

### Sample collection, measurements, and analysis

At 70 DFS (BBCH 62/63 flowers open on 2–3 racemes per plant), ten *V. faba* plants were randomly sampled from each experimental plot for measuring their physiological and biochemical responses, leaf and seed Cd content, leaf anatomical structure, growth and yield measurements as follows:

### Tissue water contents and membrane stability

Following the guidelines provided by Hayat et al. [43] and Premachandra et al. [44], the relative water content (RWC%) and membrane stability index (MSI%) in the youngest well-developed leaves were measured.

### Photosynthetic pigments and chlorophyll *a* fluorescence

Chlorophyll *a* fluorescence traits including maximum quantum yield efficiency of PSII (*F*_*v*_*/F*_*m*_) [45] and performance index (PI_ABS_) based on equal absorption [46] were calculated from measurements taken from one leaf randomly selected per plant on two different bright days using a portable Handy PEA fluorometer (Hansatech Instruments Ltd, Kings Lynn, UK).

### Osmoprotectants, total phenolic, and 2,2-diphenyl-1-picrylhydrazyl (DPPH)

Dried leaves (0.5 g) were used for determination of free proline (FPro) following Bares et al. [47] methods on dry weight basis (mg g^− 1^). Irigoyen et al. [48] method was used for extraction and determination of total soluble sugars (TSS) from dried leaves using UV–visible Spectrophotometer (UV-160 A, Shimadzu, Kyoto, Japan) at 625 nm. Total soluble protein (TSP) was determined in the dry leaves using Folin phenol as a reagent following the methodology of Singleton and Rossi [49]. Using the methanolic extract of the same dry material [50], a Folin-Ciocalteu colorimetric technique was applied to determine the total phenolic content. The DPPH-RSA %of the extract was calculated by DPPH free radical according to Lowry et al. [51].

### Oxidative stress indicators, and non-enzymatic and enzymatic antioxidants

To assay oxidative stress, hydrogen peroxide (H_2_O_2_; µmol mg^− 1^ FW) was determined as described by Patterson et al. [52]. To estimate lipid peroxidation of *V. faba* leaves, malondialdehyde (MDA; µmol mg^− 1^ FW) content was measured by the thiobarbituric acid assay as defined by Heath and Packer [53]. The ascorbic acid (AsA) and reduced glutathione (GSH; µmol g^− 1^ FW) contents in *V. faba* fresh leaf tissue were determined using the techniques outlined by Griffith [54] and Mukherjee and Choudhuri [55], respectively. Plant cells were extracted following the technique of Bradford [56] to measure enzymatic antioxidant activity (U mg^‒1^ protein). Using the guidelines provided by Yoshimura et al. [57], the APX activity was determined. CAT activity was established using the approach published by Aebi [58]. Glutathione reductase (GR) activity was determined according to Aebi [58] at 340 nm. The superoxide dismutase (SOD) activity was assessed according to Kono [59] at 540 nm.

### Leaf and seed Cd content

Using atomic absorption spectrophotometry (AAS, Perkin-Elmer Model 3300), the amounts of Cd^2+^ in leaves and seeds were determined, following the methodology outlined by Chapman and Pratt [60].

### Leaf and stem anatomical structures

Stem and leaf samples (the 3rd or 4th top internode from the stem with its leaf) were collected for anatomical observations. Following Mohamed et al. [61], the leaf and stem samples were fixed for 72 h in a formalin-acetic acid-alcohol (FAA) solution to kill and fix the tissue sample. An optical microscope (AxioPlan, Zeiss, Jena, Germany) coupled to a digital camera (Nikon DS-U3, Tokyo, Japan) was used to examine the slices of stems and leaves. Additionally, the CaseViewer 2.3 programme (3DHISTECH Ltd., Budapest, Hungary) was used to image analysis [62].

### Plant growth measurements

Plant height (cm) using a meter scale and shoot fresh weight (FW; g) using an electronic balance were measured. Branches no. plant^− 1^ and leaves number plant^− 1^ were counted. Leaf area plant^− 1^ (dm^− 2^) was measured using LI-COR (LI-3000; Lincoln NE, USA), while shoot dry weight (DW; g) was recorded after drying in a forced-air oven at 70 °C till a constant weight.

### Yield measurements

At the BBCH 77/78 stage (70–80% of pods have developed to final length), the *V. faba* green pods were commercially ready for fresh human consumption, so they were manually collected from all plants in an area of 6 m^2^ (one sowing row) in each experimental plot and then transformed to t ha^− 1^. At the harvesting stage (BBCH 96/97 full maturity stage), ten guarded plants were randomly selected from each experimental plot for yield components, i.e., pods No. plant^− 1^, and 100-seed weight (g). The dry pods from the remaining two rows (12 m^2^) in each plot were gathered, threshed, cleaned, and weighed, in addition to the seed weight from the ten plants sampled before to determine the seed yield kg per plot. The obtained values, later were converted to ton ha^− 1^ on a 13% seed moisture basis.

### Statistical data analysis

Using Microsoft Excel^®^ 2016 software, the dataset’s means ± standard error and plotted figures for the obtained variables were performed. Pre-performing the combined analysis of variance (ANOVA), the data for all variables were explored for normal distribution assumption and error variance homogeneity at α ≤ 0.05. All variables were statistically valid for further combined ANOVA without the need for data transformation. The combined statistical analysis for both studied seasons was performed based on a strip-plot ANOVA in RCBD [63], using the GenStat (version 12, VSN International Ltd., Oxford, UK) software package. The treatment means were separated based on Duncan’s test at α ≤ 0.05. Utilizing the statistical R (version 4.0.2) software tool, the PCA-biplot and Pearson heat map correlation among the selected variables were done.

## Results

### Impacts of LRE + Mt on leaf integrity and photosynthetic efficiency

The exposure to Cd and salinity stress significantly decreased the relative water content (RWC), membrane stability index (MSI), chlorophyll value (SPAD), chlorophyll a fluorescence (*F*_*v*_*/F*_*m*_ and *F*_*v*_*/F*_*0*_), and performance index (PI) of *V. faba* plants (Table 2). Nevertheless, the treatment of licorice root extract (LRE) and melatonin (Mt) not only enhanced these parameters but also mitigated the decrease caused by Cd and salt stress. Relative to control, the application of Mt resulted in an increase of RWC by 11% and 12%, MSI by 9% and 18%, SPAD by 50% and 55%, *Fv/Fm* by 10% and 13%, *Fv/F*_*0*_ by 54% and 83%, and PI by 220% and 337% at both concentrations of Mt_50_ and MT_100_, respectively (Table 2). The application of LRE alone increased the RWC, MSI, SPAD, *F*_*v*_*/F*_*m*_, *F*_*v*_*/F*_*0*_, and PI by 6%, 4%, 27%, 13%, 78%, and 317%, respectively. Whereas, the application of LRE + Mt_50_ and LRE + Mt_100_ caused an increase of 14% and 15 in RWC, 6% and 20% in MSI, 65% and 77% in SPAD, 13% and 13% in *F*_*v*_*/F*_*m*_, 82% and 82% in *F*_*v*_*/F*_*0*_, and 343% and 363% in PI, respectively (Table 2).

**Table 2:** Impacts of licorice root extract (LRE) and melatonin (Mt) on leaf integrity and photosynthetic efficiency of *V. faba* grown in Cd-contaminated saline soil

Treatment^1^		RWC (%)	MSI (%)	SPAD	F_v_/F_m_	F_v_/F_0_	PI %
LRE × Mt		**	*	**	**	**	*
-LRE	Mt_0_	64.84 ± 2.8^c^	59.63 ± 2.9^b^	30.72 ± 1.0^c^	0.748 ± 0.01^c^	3.04 ± 0.23^c^	1.89 ± 0.22^b^
	Mt_50_	72.14 ± 3.1^ab^	65.23 ± 2.5^ab^	46.17 ± 2.2^ab^	0.824 ± 0.01^b^	4.67 ± 0.44^b^	6.05 ± 0.52^a^
	Mt_100_	72.45 ± 2.6^ab^	70.13 ± 3.3^b^	47.68 ± 2.4^ab^	0.847 ± 0.00^a^	5.57 ± 0.16^a^	8.26 ± 0.76^a^
+LRE	Mt_0_	68.49 ± 2.6^ab^	61.92 ± 1.3^b^	38.98 ± 7.2^bc^	0.843 ± 0.00^a^	5.40 ± 0.13^a^	7.88 ± 0.77^a^
	Mt_50_	73.76 ± 4.3^a^	63.01 ± 2.4^a^	50.62 ± 2.3^a^	0.847 ± 0.00^a^	5.54 ± 0.11^a^	8.37 ± 1.15^a^
	Mt_100_	74.34 ± 4.3^a^	71.40 ± 4.4^a^	54.28 ± 1.3^a^	0.848 ± 0.00^a^	5.54 ± 0.09^a^	8.76 ± 0.38^a^

^**1**^Values are means ± standard error. * and ** indicate differences at *p* ≤ 0.05 and 0.01 probability level and “ns” indicates not significant difference *p* ≤ 0.05. Treatments are defined as follows: -LRE + Mt_0_ (control, no LRE or Mt foliar application), -LRE + Mt_50_ (no LRE foliar application + 50 mM Mt foliar application), -LRE + Mt_100_ (no LRE foliar application + 100 mM Mt foliar application), +LRE + Mt_0_ (3% LRE foliar application + 0 mM Mt), +LRE + Mt_50_ (3% LRE foliar application + 50 mM Mt foliar application), and + LRE + Mt_100_ (3% LRE foliar application + 100 mM Mt foliar application). RWC; relative water content, MSI; membrane stability index, SPAD; soil plant analysis development, *Fv/Fm* and *Fv/F0*; chlorophyll *a* fluorescence, and PI; performance index

### Impacts of LRE + Mt on Cd content (leaves and seeds) and oxidative stress biomarkers

The data shown in Table 3 illustrates the impact of LRE and Mt on cadmium content (leaves and seeds), oxidative stress (H_2_O_2_), and lipid peroxidation (MDA) of *V. faba* plants grown on Cd-contaminated saline soils. Cd and salinity stress considerably raised the Cd content in the leaves and seeds of *V. faba* plants, as well as the levels of H_2_O_2_ and MDA (Table 3). Mt supplementation considerably reduced Cd content, H_2_O_2_, and MDA levels, with most substantial declines were observed due to 100 (µmol g^− 1^ FW) concentration of Mt. Treatment of Mt_50_ reduced Cd content by 54% in both leaves and seeds, as well as H_2_O_2_ and MDA by 7% and 10%, respectively over the control. Whereas, Mt_100_ treatment declined leaf and seed Cd content by 64%, H_2_O_2_ by 18%, and MDA by 13% compared to the control. On the other hand, the application of LRE alone decreased leaf Cd, seed Cd, H_2_O_2,_ and MDA by 53%, 53%, 9%, and 6%, respectively. Integrative application of LRE and Mt to Cd-stressed plants grown in saline soil considerably decreased leaf Cd, seed Cd, H_2_O_2,_ and MDA with maximal alleviation observed due to the application of Mt_100_. The application of LRE + Mt_50_ and LRE + Mt_100_ resulted in an increase of 68% and 71% in leaf Cd, 68% and 71% in seed Cd, 26% and 29% in H_2_O_2_, and 18% and 26% in MDA, respectively (Table 3).

**Table 3:** Impacts of licorice root extract (LRE) and melatonin (Mt) on Cd content (leaves and seeds) and oxidative stress biomarkers of *V. faba* grown in Cd-contaminated saline soil

Treatment^1^		Leaf Cd	Seed Cd	H_2_O_2_	MDA
		mg kg^− 1^ DW		µmol mg^− 1^ FW	
LRE × Mt		**	**	*	**
-LRE	Mt_0_	10.39 ± 0.2^a^	0.64 ± 0.1^a^	1.80 ± 0.02^a^	13.68 ± 0.2^a^
	Mt_50_	4.77 ± 0.1^b^	0.30 ± 0.0^b^	1.67 ± 0.02^ab^	12.32 ± 0.2^c^
	Mt_100_	3.73 ± 0.1^c^	0.23 ± 0.1^c^	1.48 ± 0.02^bc^	11.90 ± 0.2^d^
+LRE	Mt_0_	4.89 ± 0.1^b^	0.30 ± 0.2^b^	1.63 ± 0.02^ab^	12.84 ± 0.2^b^
	Mt_50_	3.32 ± 0.1^cd^	0.21 ± 0.1^cd^	1.34 ± 0.09^c^	11.18 ± 0.2^e^
	Mt_100_	3.05 ± 0.0^d^	0.19 ± 0.0^d^	1.28 ± 0.02^c^	10.15 ± 0.2^f^

^**1**^Values are means ± standard error. * and ** indicate differences at *p* ≤ 0.05 and 0.01 probability level and “ns” indicates not significant difference *p* ≤ 0.05. Treatments are defined as follows: -LRE + Mt_0_ (control, no LRE or Mt foliar application), -LRE + Mt_50_ (no LRE foliar application + 50 mM Mt foliar application), -LRE + Mt_100_ (no LRE foliar application + 100 mM Mt foliar application), +LRE + Mt_0_ (3% LRE foliar application + 0 mM Mt), +LRE + Mt_50_ (3% LRE foliar application + 50 mM Mt foliar application), and + LRE + Mt_100_ (3% LRE foliar application + 100 mM Mt foliar application). Cd; cadmium, H_2_O_2_; hydrogen peroxide, and MDA; malondialdehyde

### Impacts of LRE + Mt on osmoprotectants, total phenolic, and DPPH

The results of total soluble sugars (TSS), free proline (FPro), total phenolic, DPPH, and total soluble proteins (TSP) in *V. faba* plants exposed to Cd and salinity-induced stress are presented in (Table 4). Under Cd and salinity-induced stress the levels of TSS, FPro, total phenolic, DPPH, and TSP in *V. faba* plants were 4.18 (mg g^− 1^ FW), 27.34 (mg g^− 1^ FW), 20.68 (mg GAE/g^− 1^ DW), 3.93% (RSA (%)), and 4.39 (mg g^− 1^ FW), respectively. Relative to control, Mt_50_ resulted in an increase of 157% in TSS, 4% in FPro, 14% in total phenolic, 44% in DPPH, and 25% in TSP. Whereas, Mt_100_ increased the levels of TSS, FPro, total phenolic, DPPH, and TSP in *V. faba* plants by 196%, 16%, 15%, 47%, and 30%, respectively over the control (Table 4). A similar impact was noticed with the LRE application. The application of LRE alone increased TSS, FPro, total phenolic, DPPH, and TSP by 113%, 5%, 4%, 22%, and 3%, respectively. Integrative application of LRE and Mt markedly increased TSS, FPro, total phenolic, DPPH, and TSP with maximal increment observed due to application of LRE + Mt_100_. The application of LRE + Mt_50_ and LRE + Mt_100_ resulted in an increase of 196% and 273% in TSS, 19% and 20% in FPro, 30% and 32% in total phenolic, and 56% and 101% in DPPH, and 37% and 46% in TSP, respectively over the control (Table 4).

**Table 4:** Impacts of licorice root extract (LRE) and melatonin (Mt) on osmoprotectants, total phenolic, and DPPH of *V. faba* grown in Cd-contaminated saline soil

Treatment ^1^		TSS	FPro	Total phenolic	TSP	DPPH (%)
		mg g^− 1^				
LRE × Mt		**	**	**	**	**
-LRE	Mt_0_	4.18 ± 0.10^e^	27.34 ± 0.32^d^	20.68 ± 0.15^e^	4.39 ± 0.05^e^	3.93 ± 0.03^e^
	Mt_50_	10.74 ± 0.30^c^	28.54 ± 0.31^c^	23.56 ± 0.21^c^	5.47 ± 0.05^c^	5.65 ± 0.04^c^
	Mt_100_	12.36 ± 0.18^b^	31.84 ± 0.30^b^	23.74 ± 0.08^c^	5.70 ± 0.05^b^	5.77 ± 0.05^c^
+LRE	Mt_0_	8.90 ± 0.30^d^	28.60 ± 0.28^c^	21.48 ± 0.09^d^	4.52 ± 0.05^f^	4.81 ± 0.03^d^
	Mt_50_	12.36 ± 0.30^b^	32.56 ± 0.30^ab^	26.86 ± 0.12^b^	6.02 ± 0.05^b^	6.12 ± 0.03^b^
	Mt_100_	14.09 ± 0.42^a^	32.86 ± 0.33^a^	27.37 ± 0.14^a^	6.41 ± 0.05^a^	7.91 ± 0.03^a^

^**1**^Values are means ± standard error. * and ** indicate differences at *p* ≤ 0.05 and 0.01 probability level and “ns” indicates not significant difference *p* ≤ 0.05. Treatments are defined as follows: -LRE + Mt_0_ (control, no LRE or Mt foliar application), -LRE + Mt_50_ (no LRE foliar application + 50 mM Mt foliar application), -LRE + Mt_100_ (no LRE foliar application + 100 mM Mt foliar application), +LRE + Mt_0_ (3% LRE foliar application + 0 mM Mt), +LRE + Mt_50_ (3% LRE foliar application + 50 mM Mt foliar application), and + LRE + Mt_100_ (3% LRE foliar application + 100 mM Mt foliar application). TSS; total soluble sugars, FPro; free proline, TSP; total soluble proteins, and DPPH; 2, 2-diphenyl-1-picrylhydrazyl

### Impacts of LRE + Mt on non-enzymatic and enzymatic antioxidants

Table 5 shows how LRE + Mt affects non-enzymatic [ascorbic acids (AsA) and glutathione (GSH)] and enzymatic [ascorbate peroxidase (APX), catalase (CAT), glutathione reductase (GR), and superoxide dismutase (SOD)] antioxidants of *V. faba* plants grown in Cd-contaminated saline soil. The content of AsA, GSH, and the activity of APX, CAT, GR, and SOD in leaves of *V. faba* plants enhanced by application of Mt, compared to the control, with an increase of 17% and 26% in AsA, 15% and 26% in GSH, 13% and 55% in APX, 9% and 11% in CAT, 45% and 64% in GR, and 17% and 21% in SOD. A more or less similar impact was noticed with the LRE application. The application of LRE alone raised AsA, GSH, APX, CAT, GR, and SOD by 14%, 17%, 39%, 3%, 5%, and 10%, respectively (Table 5). In Cd-contaminated saline soil, treatment of LRE + Mt_50_ and LRE + Mt_100_ resulted in a greater rise in non-enzymatic and enzymatic antioxidants of *V. faba* than the control, with higher increment observed due to application of LRE + Mt_100_. The application of LRE + Mt_50_ and LRE + Mt_100_ resulted in an increase of 50% and 76% in AsA, 50% and 75% in GSH, 91% and 146% in APX, 12% and 15% in CAT, 70% and 76% in GR, and 26% and 34% in SOD, respectively over the control (Table 5).

**Table 5:** Impacts of licorice root extract (LRE) and melatonin (Mt) on non-enzymatic and enzymatic antioxidants of *V. faba* grown in Cd-contaminated saline soil

Treatment^1^		AsA	GSH	APX	CAT	GR	SOD
		(µmol g^− 1^ FW)		(U mg^‒1^ protein)			
LRE × Mt		**	**	**	**	**	**
-LRE	Mt_0_	2.42 ± 0.04^e^	2.43 ± 0.04^e^	5.38 ± 0.09^f^	7.21 ± 0.12^e^	11.48 ± 0.30^f^	10.27 ± 0.15^f^
	Mt_50_	2.82 ± 0.04^cd^	2.79 ± 0.08^d^	6.09 ± 0.11^e^	7.84 ± 0.12^c^	16.62 ± 0.30^d^	12.02 ± 0.15^d^
	Mt_100_	3.05 ± 0.04^c^	3.07 ± 0.05^c^	8.35 ± 0.09^c^	7.99 ± 0.12^bc^	18.86 ± 0.30^c^	12.44 ± 0.15^c^
+LRE	Mt_0_	2.77 ± 0.07^d^	2.84 ± 0.04^cd^	7.48 ± 0.07^d^	7.44 ± 0.12^d^	12.11 ± 0.30^e^	11.30 ± 0.15^e^
	Mt_50_	3.63 ± 0.04^b^	3.64 ± 0.04^b^	10.30 ± 0.08^b^	8.08 ± 0.12^b^	19.50 ± 0.30^b^	12.96 ± 0.15^b^
	Mt_100_	4.27 ± 0.11^a^	4.26 ± 0.11^a^	13.26 ± 0.12^a^	8.32 ± 0.12^a^	20.23 ± 0.30^a^	13.80 ± 0.15^a^

^**1**^Values are means ± standard error. * and ** indicate differences at *p* ≤ 0.05 and 0.01 probability level and “ns” indicates not significant difference *p* ≤ 0.05. Treatments are defined as follows: -LRE + Mt_0_ (control, no LRE or Mt foliar application), -LRE + Mt_50_ (no LRE foliar application + 50 mM Mt foliar application), -LRE + Mt_100_ (no LRE foliar application + 100 mM Mt foliar application), +LRE + Mt_0_ (3% LRE foliar application + 0 mM Mt), +LRE + Mt_50_ (3% LRE foliar application + 50 mM Mt foliar application), and + LRE + Mt_100_ (3% LRE foliar application + 100 mM Mt foliar application). AsA; ascorbic acids, GSH; glutathione, APX; ascorbate peroxidase, CAT; catalase, GR; glutathione reductase, and SOD; superoxide dismutase

### Impacts of LRE + Mt on *V. faba*’s leaf anatomical structure

**Fig. 1:**
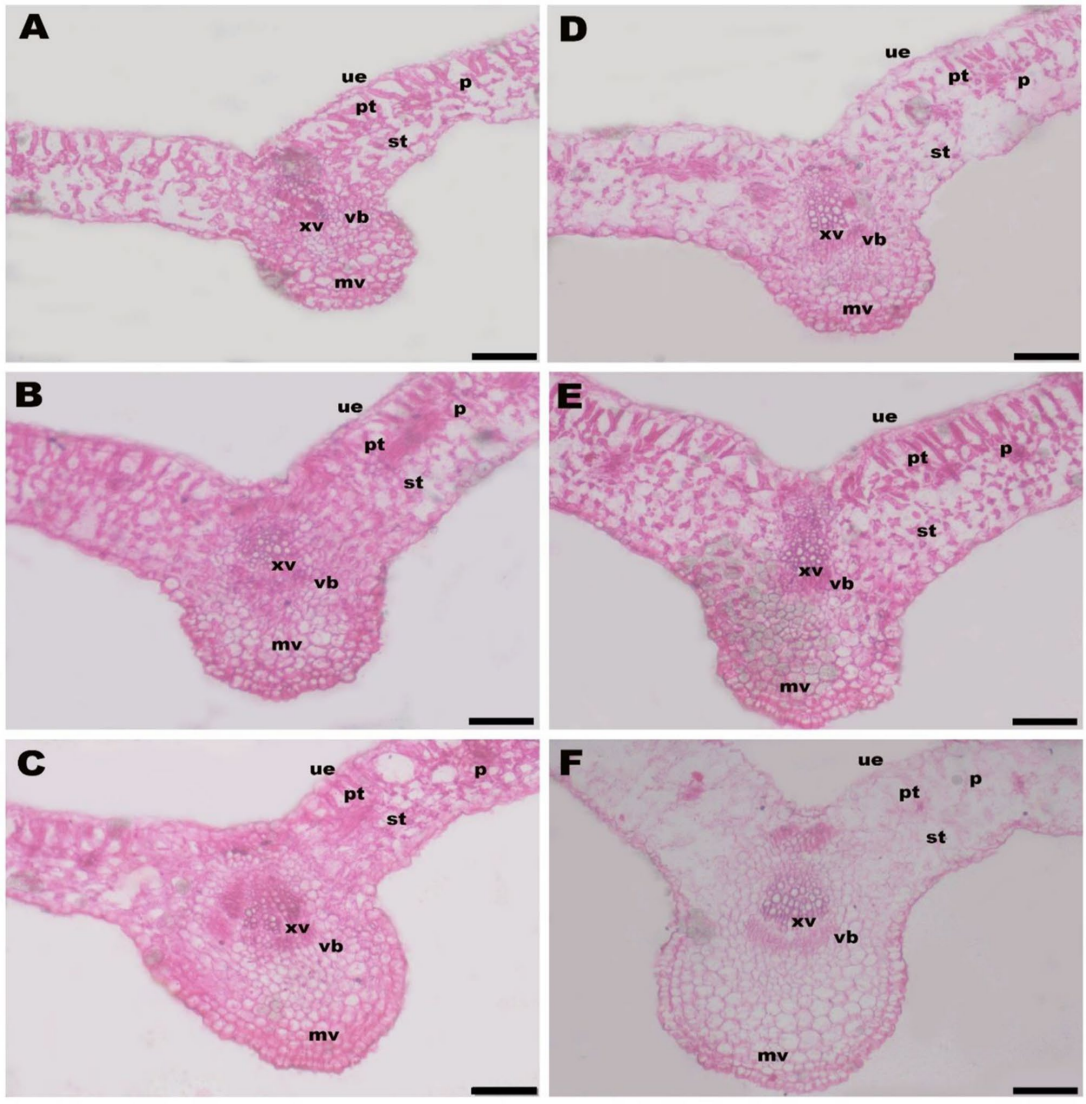
Cross transections in *V. faba* leaf affected by licorice root extract (LRE) and melatonin (Mt). **A**= -LRE + Mt_0_ (control, no LRE or Mt foliar application), **B**= -LRE + Mt_50_ (no LRE foliar application + 50 mM Mt foliar application), **C**= -LRE + Mt_100_ (no LRE foliar application + 100 mM Mt foliar application), **D**= +LRE + Mt_0_ (3% LRE foliar application + 0 mM Mt), E = + LRE + Mt_50_ (3% LRE foliar application + 50 mM Mt foliar application), and F + LRE + Mt_100_ (3% LRE foliar application + 100 mM Mt foliar application). ue; upper epidermis, pt; plastid parenchyma tissue, st; sponge parenchyma tissue, xv; xylem vessels, vb; vascular bundle of the midvein, and mv; midvein. Scale bar = 200 μm

The supplementations with LRE + Mt had a noticeable effect (*p* ≤ 0.05 and *p* ≤ 0.01) on the leaf anatomical structure i.e., midvein height (MH), midvein width (MW), midvein vascular bundle height (MVBH), midvein vascular bundle width (MVBW), leaf blade thickness (LBT), spongy tissue thickness (STT), and plastid thickness (PT) of *V. faba* plants grown on Cd-contaminated saline soil (Table 6; Fig. 1). Under Cd and salinity stress, MH, MW, MVBH, MVBW, LBT, STT, and PT in *V. faba* plants were 659.7 μm, 553.5 μm, 279.2 μm, 147.7 μm, 355.2 μm, 194.7 μm, and 95.7 μm, respectively. Compared to the control, Mt_50_ treatment resulted in an increase of 11% in MH, 1% in MW, 19% in MVBH, 36% in MVBW, 4% in LBT, 2% in STT, and 3% in PT. Whereas, Mt_100_ increased the MH, MW, MVBH, MVBW, LBT, STT, and PT in *V. faba* plants by 29%, 26%, 6%, 36%, 6%, 2%, and 10%, respectively over the control (Table 6). A more or less similar impact was noticed with LRE application. LRE treatment alone improved MH, MW, MVBH, MVBW, LBT, STT, and PT by 7%, 17%, 11%, 9%, 3%, 0%, 3%, respectively. Integrative application of LRE and Mt markedly increased MH, MW, MVBH, MVBW, LBT, STT, and PT with maximal increment observed due to using of LRE + Mt_100_. In comparison to the control, the application of LRE + Mt_50_ and LRE + Mt_100_ led to an increase of 35% and 44% in MH, 27% and 29% in MW, 4% and 5% in MVBH, 41% and 41% in MVBW, 7% and 11% in LBT, 3% and 9% in STT, and 10% and 35% in PT (Table 6).

**Table 6:** Impacts of licorice root extract (LRE) and melatonin (Mt) on leaf anatomical structure of *V. faba* grown in Cd-contaminated saline soil

Treatment^1^		Midvein height	Midvein width	Midvein Vascular bundle height	Midvein vascular bundle width	Leaf blade thickness	Spongy tissue thickness	Plastid thickness
				(µm)				
LRE × Mt		**	**	NS	**	**	**	**
-LRE	Mt_0_	659.7 ± 7.7^f^	553.5 ± 1.6^b^	279.2 ± 9.3^a^	147.7 ± 2.2^b^	355.2 ± 18.5^d^	194.7 ± 2.3^b^	95.7 ± 2.2^d^
	Mt_50_	729.7 ± 4.4^d^	556.8 ± 9.3^b^	225.8 ± 9.3^a^	201.0 ± 4.0^ab^	368.5 ± 2.9^bc^	198.0 ± 3.8^ab^	99.0 ± 1.9^c^
	Mt_100_	849.7 ± 7.7^c^	696.8 ± 4.5^a^	295.8 ± 4.9^a^	201.0 ± 4.4^ab^	375.2 ± 17.0^bc^	198.0 ± 2.0^ab^	105.7 ± 2.2^b^
+LRE	Mt_0_	703.0 ± 3.9^e^	650.2 ± 2.6^a^	309.2 ± 4.4^a^	161.0 ± 6.3^ab^	365.2 ± 2.2^cd^	194.7 ± 2.3^b^	99.0 ± 0.4^c^
	Mt_50_	893.0 ± 3.9^b^	703.5 ± 7.5^a^	265.8 ± 4.4^a^	207.7 ± 13.8^a^	378.5 ± 8.8^b^	201.3 ± 2.3^ab^	105.7 ± 4.2^b^
	Mt_100_	953.0 ± 1.3^a^	713.5 ± 4.0^a^	269.2 ± 10.6^a^	207.7 ± 8.4^a^	395.2 ± 3.0^a^	211.3 ± 2.3^a^	129.0 ± 1.9^a^

^**1**^Values are means ± standard error. * and ** indicate differences at *p* ≤ 0.05 and 0.01 probability level and “ns” indicates not significant difference *p* ≤ 0.05. Treatments are defined as follows: -LRE + Mt_0_ (control, no LRE or Mt foliar application), -LRE + Mt_50_ (no LRE foliar application + 50 mM Mt foliar application), -LRE + Mt_100_ (no LRE foliar application + 100 mM Mt foliar application), +LRE + Mt_0_ (3% LRE foliar application + 0 mM Mt), +LRE + Mt_50_ (3% LRE foliar application + 50 mM Mt foliar application), and + LRE + Mt_100_ (3% LRE foliar application + 100 mM Mt foliar application)

### Impacts of LRE + Mt on *V. faba’s* stem anatomical structure

**Fig. 2:**
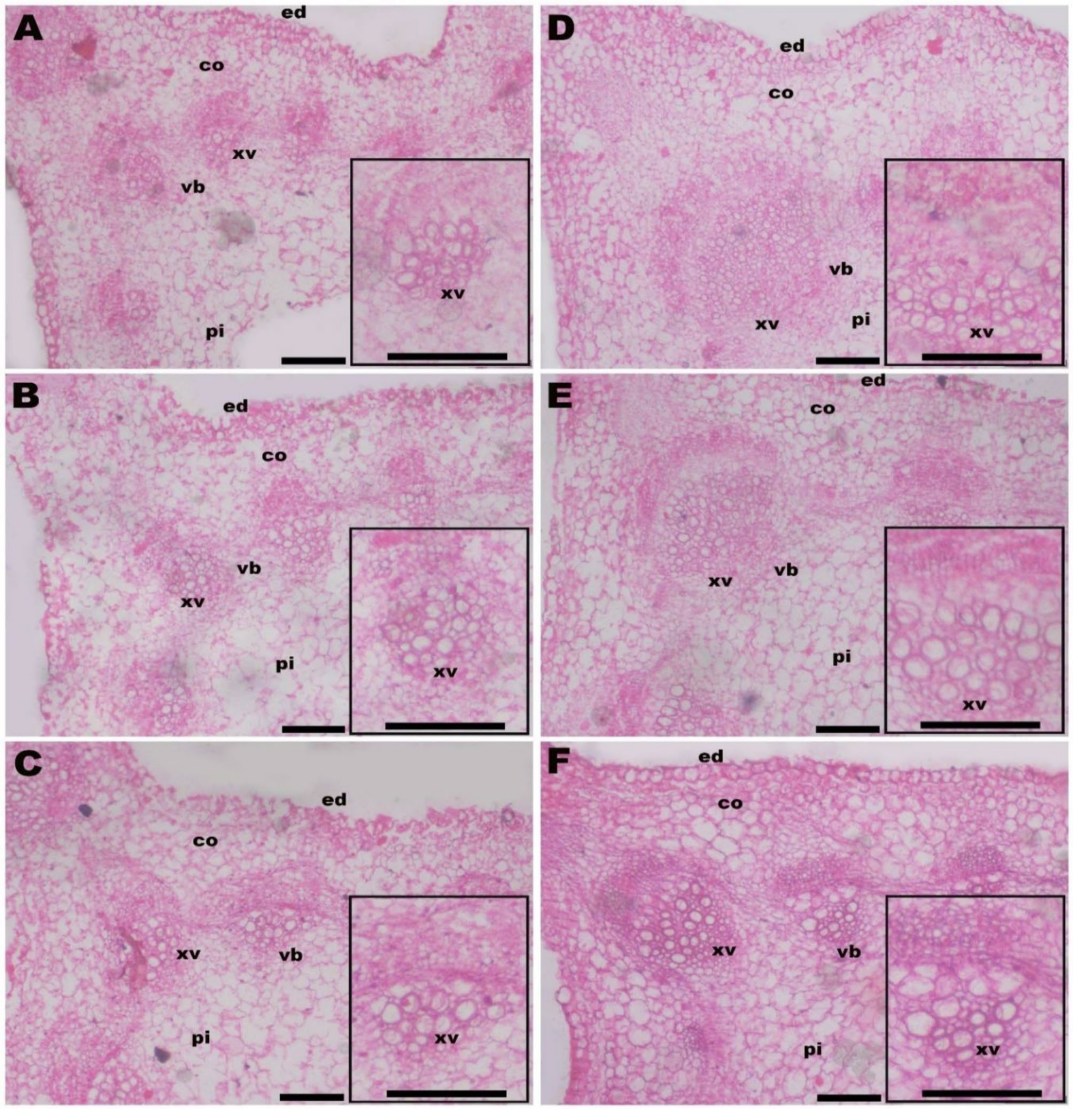
Cross transections in *V. faba* stem affected by licorice root extract (LRE) and melatonin (Mt). **A**= -LRE + Mt_0_ (control, no LRE or Mt foliar application), **B**= -LRE + Mt_50_ (no LRE foliar application + 50 mM Mt foliar application), **C**= -LRE + Mt_100_ (no LRE foliar application + 100 mM Mt foliar application), **D** = + LRE + Mt_0_ (3% LRE foliar application + 0 mM Mt), E = + LRE + Mt_50_ (3% LRE foliar application + 50 mM Mt foliar application), and F + LRE + Mt_100_ (3% LRE foliar application + 100 mM Mt foliar application). vb; vascular bundle, pi; pith, xv; xylem vessels, co; cortex, and ed; epidermis layer. Small rectangles are vascular bundles. Scale bar = 200 μm

Table 7; Fig. 2 demonstrate the significant impact of integrative application with LRE + Mt on stem anatomical structure i.e., stem width (SW), stem length (SL), xylem vessel thickness (XVT), vascular bundle diameter (VBD), xylem vessel diameter (XVD), cortex thickness (CT), pith width (PW), and pith length (PL) of *V. faba* plants subjected to Cd and salinity-induced stress. When exposed to stress caused by Cd and salinity, the measurements of SW, SL, XVT, VBD, XVD, CT, PW, and PL were 2476 μm, 3132 μm, 20.83 μm, 284.3 μm, 77.5 μm, 176.5 μm, 1615.8 μm, and 1940 μm, respectively. Relative to control, Mt_50_ treatment resulted in an increase of 28% in SW, 29% in SL, 18% in XVT, 15% in VBD, 19% in XVD, 24% in CT, 40% in PW, and 49% in PL. Whereas, Mt_100_ increased the measurements of SW, SL, XVT, VBD, XVD, CT, PW, and PL by 47%, 59%, 26%, 23%, 8%, 42%, 63%, and 98%, respectively as compared to control (Table 7). The application of LRE alone increased SW, SL, XVT, VBD, XVD, CT, PW, and PL by 28%, 29%, 2%, 6%, 0%, 11%, 32%, and 51%, respectively. Integrative application of LRE and Mt markedly increased the measurements of SW, SL, XVT, VBD, XVD, CT, PW, and PL with maximal improvements were observed due to using of LRE + Mt_100_. Compared to the control, LRE + Mt_50_ and LRE + Mt_100_ increased SW by 65% and 74%, SL by 90% and 98%, XVT by 26% and 35%, VBD by 32% and 44%, XVD by 6% and 21%, CT by 42% and 85%, PW by 78% and 99%, and PL by 104% and 106%, respectively (Table 7).

**Table 7:** Impacts of licorice root extract (LRE) and melatonin (Mt) on stem anatomical structure of *V. faba* grown in Cd-contaminated saline soil

Treatment^1^		Stem width	Stem length	Xylem vessel thickness	Vascular bundle diameter	Xylem vessel diameter	Cortex thickness	Pith width	Pith length
				(µm)					
LRE × Mt		**	**	**	**	**	**	**	**
-LRE	Mt_0_	2476 ± 21.5^e^	3132 ± 3.1^e^	20.83 ± 0.7^c^	284.3 ± 5.3^d^	77.5 ± 0.7^b^	176.5 ± 0.7^e^	1615.8 ± 11.1^f^	1940 ± 19.2^c^
	Mt_50_	3176 ± 26.7^d^	4049 ± 26.5^d^	24.50 ± 0.8^b^	326.0 ± 9.1^cd^	62.5 ± 0.7^c^	218.2 ± 4.3^c^	2257.5 ± 3.4^d^	2882 ± 27.5^b^
	Mt_100_	3635 ± 45.8^c^	4965 ± 26.5^c^	26.17 ± 0.7^ab^	351.0 ± 9.1^bc^	83.5 ± 0.7^a^	251.5 ± 1.9^b^	2640.8 ± 46.1^c^	3840 ± 26.5^a^
+LRE	Mt_0_	3176 ± 42.4^d^	4049 ± 26.5^d^	21.17 ± 0.7^c^	301.0 ± 0.4^d^	77.5 ± 0.7^b^	196.5 ± 3.2^d^	2132.5 ± 50.9^e^	2923 ± 26.5^b^
	Mt_50_	4093 ± 26.7^b^	5965 ± 69.8^a^	26.17 ± 0.7^ab^	376.0 ± 0.4^ab^	61.5 ± 0.7^c^	251.5 ± 0.7^b^	2882.5 ± 3.4^b^	3965 ± 32.2^a^
	Mt_100_	4301 ± 26.7^a^	6215 ± 26.5^b^	28.17 ± 0.7^a^	409.3 ± 5.3^a^	72.5 ± 0.8^b^	326.5 ± 0.7^a^	3215.8 ± 26.6^a^	4006 ± 9.6^a^

^**1**^Values are means ± standard error. * and ** indicate differences at *p* ≤ 0.05 and 0.01 probability level and “ns” indicates not significant difference *p* ≤ 0.05. Treatments are defined as follows: -LRE + Mt_0_ (control, no LRE or Mt foliar application), -LRE + Mt_50_ (no LRE foliar application + 50 mM Mt foliar application), -LRE + Mt_100_ (no LRE foliar application + 100 mM Mt foliar application), +LRE + Mt_0_ (3% LRE foliar application + 0 mM Mt), +LRE + Mt_50_ (3% LRE foliar application + 50 mM Mt foliar application), and + LRE + Mt_100_ (3% LRE foliar application + 100 mM Mt foliar application)

### Impacts of LRE + Mt on growth characteristics of *V. faba*

The growth characteristics viz., plant height (PH), plant branch number (PBN), plant leaf number (PLN), plant leaf area (LAP), shoot FW (SFW) and Shoot DW (SDW) of *V. faba* plants grown in Cd-contaminated saline soil positively influenced (*p* ≤ 0.05 and *p* ≤ 0.01) by integrative supplementation with LRE + Mt (Table 8). Under Cd and salinity-induced stress, the growth attributes PH, PBN, PLN, LAP, SFW, and SDW of *V. faba* plants were 69 cm, 3.17, 52.7, 62.8 dm^-2^, 238 g, and 95 g, respectively. Cd and salinity stress had a significant impact on the growth attributes. However, Mt application enhanced these parameters and also mitigated the reduction caused by Cd and salinity stress. Mt_50_ and Mt_100_ increased PH by 40% and 43%, PBN by 52% and 58%, PLN by 65% and 68%, LAP by 55% and 132%, SFW by 67% and 131%, and SDW by 60% and 131%, respectively, over the control. The application of LRE had a more or less similar impact, with LRE increasing the growth attributes PH, PBN, PLN, LAP, SFW, and SDW by 35%, 32%, 41%, 64%, 95%, and 95%, respectively, over the control (Table 8). The integrative application of LRE + Mt improved the growth characteristics of plants exposed to Cd and salinity-induced stress, with maximal improvement observed in those supplemented by LRE + Mt_100_. LRE + Mt_50_ and LRE + Mt_100_ increased PH by 43% and 51%, PBN by 68% and 84%, PLN by 83% and 106%, LAP by 140% and 172%, SFW by 139% and 172%, and SDW by 139% and 172%, respectively, as compared to the control (Table 8).

**Table 8:** Impacts of licorice root extract (LRE) and melatonin (Mt) on growth characteristics of *V. faba* grown in Cd-contaminated saline soil

Treatment^1^		Plant height (cm)	Branches no. plant^− 1^	Leaves no.	Leaf area plant^− 1^ (dm^− 2^)	Shoot FW (g)	Shoot DW (g)
LRE × Mt		*	NS	*	*	*	*
-LRE	Mt_0_	69.0 ± 2.91^b^	3.17 ± 0.17^a^	52.7 ± 3.21^e^	62.8 ± 1.0^d^	238 ± 3.9^d^	95.0 ± 1.5^d^
	Mt_50_	96.7 ± 7.88^a^	4.83 ± 0.45^a^	86.7 ± 10.2^c^	97.3 ± 8.8^c^	398 ± 33.3^c^	152.0 ± 13.3^c^
	Mt_100_	98.7 ± 2.33^a^	5.00 ± 0.79^a^	88.3 ± 10.0^bc^	145.4 ± 3.4^b^	550 ± 38.6^b^	219.9 ± 35.4^ab^
+LRE	Mt_0_	93.0 ± 6.70^a^	4.17 ± 0.31^a^	74.5 ± 4.30^d^	102.8 ± 1.8^c^	464 ± 6.7^c^	185.7 ± 2.7^c^
	Mt_50_	99.0 ± 5.14^a^	5.33 ± 0.83^a^	96.2 ± 13.6^b^	150.5 ± 3.2^ab^	569 ± 11.9^b^	227.5 ± 4.8^ab^
	Mt_100_	104.3 ± 4.43^a^	5.83 ± 0.33^a^	108.7 ± 11.5^a^	171.1 ± 0.9^a^	647 ± 3.6^a^	258.7 ± 1.4^a^

^**1**^Values are means ± standard error. * and ** indicate differences at *p* ≤ 0.05 and 0.01 probability level and “ns” indicates not significant difference *p* ≤ 0.05. Treatments are defined as follows: -LRE + Mt_0_ (control, no LRE or Mt foliar application), -LRE + Mt_50_ (no LRE foliar application + 50 mM Mt foliar application), -LRE + Mt_100_ (no LRE foliar application + 100 mM Mt foliar application), +LRE + Mt_0_ (3% LRE foliar application + 0 mM Mt), +LRE + Mt_50_ (3% LRE foliar application + 50 mM Mt foliar application), and + LRE + Mt_100_ (3% LRE foliar application + 100 mM Mt foliar application), FW; fresh weight, and DW; dry weight

### Impacts of LRE + Mt on yield-related traits of *V. faba*

Cd and salinity-induced stress significantly affected the fruit yield characteristics of *V. faba* plants (Table 9). Yield-related traits such as green pods yield (GPY), Pods No plant^-1^ (PNP), 100 seed weight (SW), and Seed yield (SY) of *V. faba* plants exposed to stress conditions were 8.26 t ha^-1^, 9.17, 86.8 g, 2.30 t ha^-1^, respectively. However, plants treated with Mt increased these parameters and also mitigated the decline induced by Cd and salinity stress. Mt_50_ and Mt_100_ increased GPY by 69% and 130%, PNP by 122% and 136%, SW by 5% and 11%, and SY by 20% and 35%, respectively, over the control. Additionally, LRE increased GPY, PNP, SW, and SY by 105%, 91%, 18%, and 27%, respectively over the control (Table 9). The integrative application of LRE + Mt improved the yield characteristics of plants exposed to Cd and salinity stress, with maximal improvement observed in those supplemented by LRE + Mt_100_. The application of LRE + Mt_50_ and LRE + Mt_100_ resulted in an increase of 152% and 183% in GPY, 136% and 140% in PNP, 13% and 22% in SW, and 53% and 68% in SY, respectively as compared to control (Table 9).

**Table 9:** Impacts of licorice root extract (LRE) and melatonin (Mt) on yield-related traits of *V. faba* grown in Cd-contaminated saline soil

Treatment^1^		Green pods yield (t ha^− 1^)	Pods no plant^− 1^	100 seed weight (g)	Seed yield (t ha^− 1^)
LRE × Mt		**	**	*	**
-LRE	Mt_0_	8.26 ± 0.21^f^	9.17 ± 0.7^c^	86.8 ± 1.3^b^	2.30 ± 0.02^f^
	Mt_50_	13.92 ± 0.20^e^	20.33 ± 0.2^a^	91.2 ± 2.7^ab^	2.75 ± 0.00^e^
	Mt_100_	19.01 ± 0.45^c^	21.67 ± 1.6^a^	96.2 ± 1.4^ab^	3.11 ± 0.20^c^
+LRE	Mt_0_	16.97 ± 0.43^d^	17.50 ± 1.0^b^	102.8 ± 1.6^ab^	2.92 ± 0.01^d^
	Mt_50_	20.81 ± 0.26^b^	21.67 ± 1.6^a^	98.5 ± 1.4^a^	3.52 ± 0.10^b^
	Mt_100_	23.36 ± 0.34^a^	22.00 ± 0.7^a^	106.0 ± 1.6^a^	3.87 ± 0.03^a^

^**1**^Values are means ± standard error. * and ** indicate differences at *p* ≤ 0.05 and 0.01 probability level and “ns” indicates not significant difference *p* ≤ 0.05. Treatments are defined as follows: -LRE + Mt_0_ (control, no LRE or Mt foliar application), -LRE + Mt_50_ (no LRE foliar application + 50 mM Mt foliar application), -LRE + Mt_100_ (no LRE foliar application + 100 mM Mt foliar application), +LRE + Mt_0_ (3% LRE foliar application + 0 mM Mt), +LRE + Mt_50_ (3% LRE foliar application + 50 mM Mt foliar application), and + LRE + Mt_100_ (3% LRE foliar application + 100 mM Mt foliar application)

### Correlation analysis between the studied traits

**Fig. 3:**
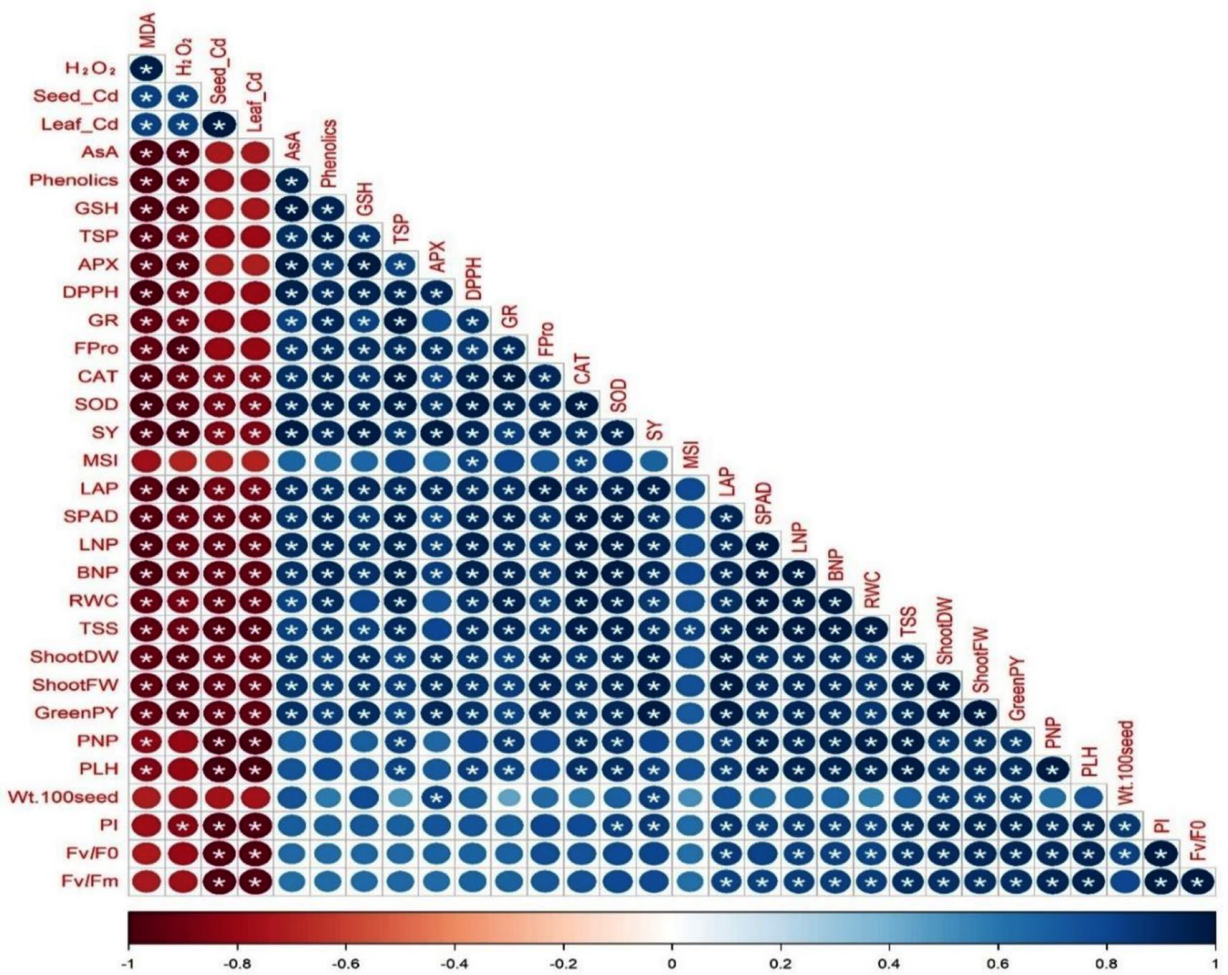
Graph of Pearson’s correlation analysis among the different studied parameters. The colors represent variations in the obtained data. * indicates the significant at *p* ≤ 0.05. The parameters photosystem II quantum efficiency, performance index, 100 seed weight, plant height, pods number plant^-1^, green pods yield, shoot fresh weight, shoot dry weight, total soluble sugars, relative water content, branch number plant^-1^, leaf number plant^-1^, soil plant analysis development, leaf area plant^-1^, membrane stability index, seed yield, superoxide dismutase, catalase, free proline, glutathione reductase, 2,2-diphenyl-1-picrylhydrazyl, ascorbate peroxidase, total soluble proteins, glutathione, and ascorbic acid are abbreviated as (*F*_*v*_*/F*_*0*_ and *F*_*v*_*/F*_*m*_), PI, Wt.100-seed, PLH, PNP, GreenPY, ShootFW, ShootDW, TSS, RWC, BNP, LNP, SPAD, LAP, MSI, SY, SOD, CAT, FPro, GR, DPPH, APX, TSP, GSH, and AsA, respectively. Values based on the average of 2022/23 and 2023/24 winter seasons

Figure 3 depicts the relationships between different variables of *V. faba* plants impacted by the integrative application of LRE and Mt under Cd-contaminated saline soil conditions. The current findings showed that greenPY and SY were positively correlated (*p*-value ≤ 0.05) with RWC (0.867 and 0.857), SPAD (0.900 and 0.903), PI (0.945 and 0.846), TSS (0.928 and 0.894), FPro (0.919 and 0.928), phenolics (0.849 and 0.926), DPPH (0.871 and 0.927), TSP (0.825 and 0.876), AsA (0.825 and 0.876), GSH (0.902 and 0.978), APX (0.912 and 0.975), GR (0.813 and 0.835), SOD (0.927 and 0.954) in leaves, PLH (0.903 and 0.835), BNP (0.918 and 0.921), LNP (0.922 and 0.931), LAP (0.971 and 0.964), shootFW (0.997 and 0.961), shootDW (0.997 and 0.962), PNP (0.872 and 0.799), and wt.100seed (0.875 and 0.836), respectively. Meanwhile, GreenPY and SY were negatively correlated with leaf Cd (-0.921 and − 0.842), H_2_O_2_ (-0.959 and − 0.985), MDA (0.920 and − 0.971) and seed Cd (-0.922 and − 0.842), respectively.

**Figure 4:**
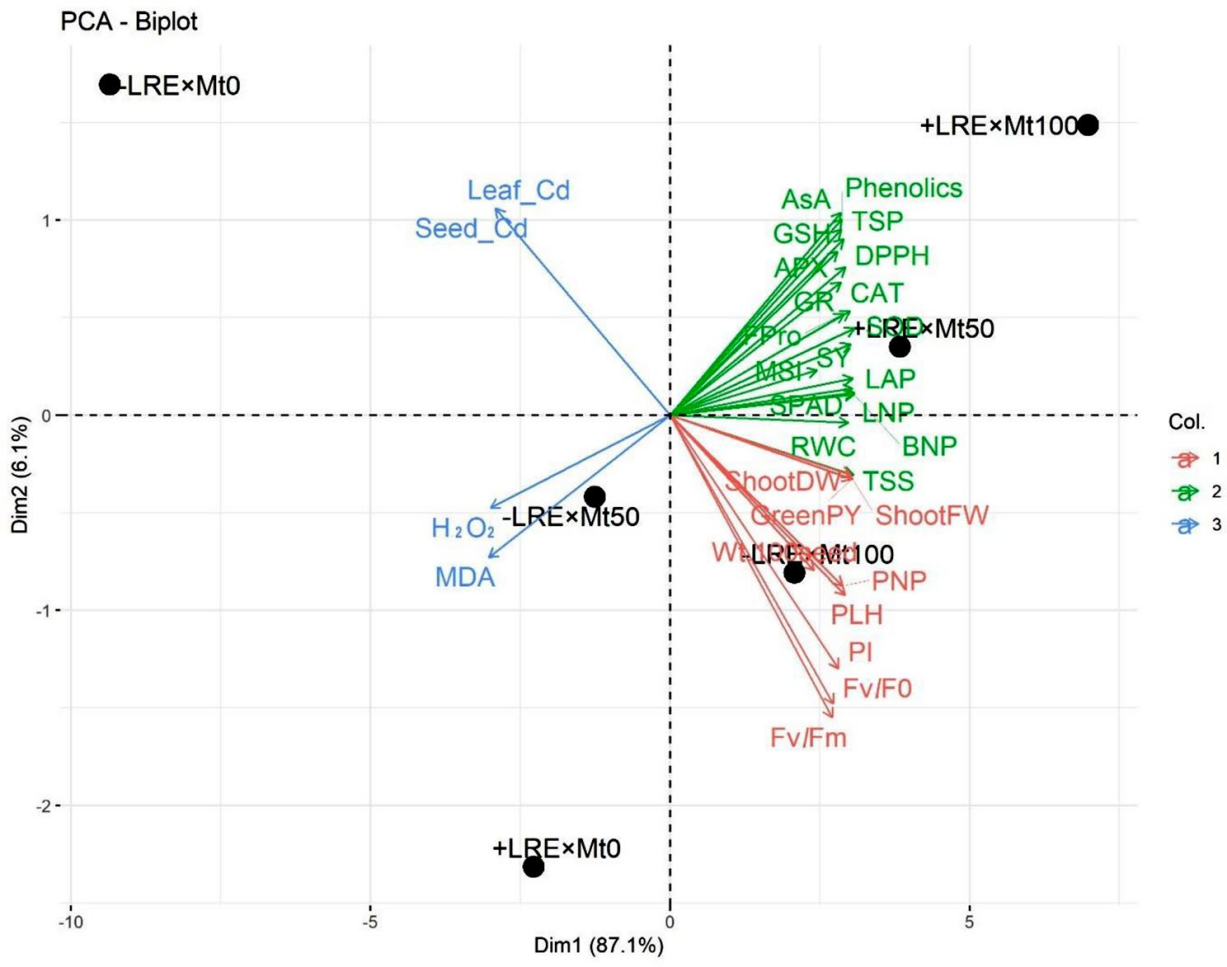
Biplot graph of the treatments and parameters examined, illustrating the two principal component analyses (Dim1 and Dim2) in *V. faba* plants affected by the combined application of licorice root extract (LRE) and melatonin (Mt) under cadmium-contaminated saline soil conditions. Variables are color-coded based on K-means clustering. Treatments are defined as follows: -LRE + Mt_0_ (control, no LRE or Mt foliar application), -LRE + Mt_50_ (no LRE foliar application + 50 mM Mt foliar application), -LRE + Mt_100_ (no LRE foliar application + 100 mM Mt foliar application), +LRE + Mt_0_ (3% LRE foliar application + 0 mM Mt), +LRE + Mt_50_ (3% LRE foliar application + 50 mM Mt foliar application), and + LRE + Mt_100_ (3% LRE foliar application + 100 mM Mt foliar application). The parameters photosystem II quantum efficiency, performance index, 100 seed weight, plant height, pods number plant^-1^, green pods yield, shoot fresh weight, shoot dry weight, total soluble sugars, relative water content, branch number plant^-1^, leaf number plant^-1^, soil plant analysis development, leaf area plant^-1^, membrane stability index, seed yield, superoxide dismutase, catalase, free proline, glutathione reductase, 2,2-diphenyl-1-picrylhydrazyl, ascorbate peroxidase, total soluble proteins, glutathione, and ascorbic acid are abbreviated as (*F*_*v*_*/F*_*0*_ and *F*_*v*_*/F*_*m*_), PI, Wt.100-seed, PLH, PNP, GreenPY, ShootFW, ShootDW, TSS, RWC, BNP, LNP, SPAD, LAP, MSI, SY, SOD, CAT, FPro, GR, DPPH, APX, TSP, GSH, and AsA, respectively. Values based on the average of 2022/23 and 2023/24 winter seasons

Figure 4 depicts a principal component analysis (PCA) biplot that examines the high variations caused by Cd-contaminated saline soil conditions and the integrative application of LRE and Mt on the studied variables. Dim 1 and Dim 2 (PCA-diminution 1 and 2, respectively) explored 87.1% and 6.1% variability of data, respectively. The large variability between -LRE + Mt_0_, -LRE + Mt_50_, -LRE + Mt_100_, +LRE + Mt_0_, +LRE + Mt_50_, and + LRE + Mt_100_ indicated the role of LRE + Mt in enhancing growth, physio-biochemistry, and yield traits of *V. faba* under Cd-contaminated saline soil conditions. The integrative application of LRE and Mt increased total phenolics, LAP, LNP, BNP, MSI, RWC, FPro, TSS, SPAD, TSP, AsA, GSH, CAT, SOD, GR, APX, DPPH, and SY. The PCA biplot revealed that the aforementioned variables were associated with LRE + Mt_50_, and LRE + Mt_100_, which had a positive influence on the growth and yield and growth of *V. faba* plants under Cd-contaminated saline soil conditions. Additionally, the PCA biplot indicated that the variables shoot (FW), shoot (DW), *F*_*v*_*/F*_*m*_, *F*_*v*_*/F*_*0*_, PI, PNP, PLH, wt.100 seed, and green yield (PY) that clustered together (Mt_100_) were also promoted by Mt. Whereas, parameters of leaf Cd, seed Cd, H_2_O_2_, and MDA content were associated with Mt_50_. Therefore, the integrative application of LRE and Mt has an important role in boosting the growth, physio-biochemistry, and yield of *V. faba* under Cd-contaminated saline soil conditions.

## Discussion

Cadmium is a heavy metal that plays no critical or beneficial role in plant growth and development [64], and it has detrimental consequences such as inhibited growth and developmental impairments, ultimately leading to reduced crop yields and quality [21, 65]. It is on the rise in the environment as a result of both anthropogenic activities and natural causes. This raises substantial apprehension regarding the safety of our food and its impact on human health [66].

Exposure to Cd toxicity had a considerable negative impact on cell membranes, and the severity of this damage was intensified with salinity stress. This reduced RWC and MSI levels in plant leaves (Table 2). Our findings align with the majority of studies, indicating that these negative effects could be due to excessive ROS production. These ROS interact with various components within the cells, including nucleic acids, lipids, and proteins, causing lipid peroxidation, compromising membrane integrity, deactivating enzymes, and disrupting the functional structure of membrane transporters. This sequence of events ultimately affects cell viability and function [67–69]. However, our research demonstrated that the supplementation with LRE + Mt mitigated the Cd-induced oxidative damage, protected cell membrane permeability, maintained water uptake and preserved cell water content [70].

Photosynthesis is a crucial physiological process in plants profoundly impacts their growth and development. Cd is widely recognized for its ability to hinder photosynthesis and decline the leaf photosynthetic efficiency of *V. faba* plants (Table 2). Our findings align with numerous previous studies indicating that photosynthesis is restrained when plants subjected to Cd stressful conditions. This inhibition occurs through affecting key synthesis enzymes like proto-chlorophyllide reductase and d-aminolevulinic acid dehydratase [71, 72], also reducing RuBP Case activity through damaging its structure by the substitution of Mg ions, and altering its activity towards oxygenation reactions [73]. Additionally, Cd influences the water-oxidizing complex of PSII by substituting Ca^2+^ in Ca/Mn clusters within the oxygen-evolving centers [74]. Furthermore, it antagonizes the uptake of Mg, Fe, K and P from soil, consequently hindering the formation of leaf porphyrin rings [21, 24, 25, 75]. However, these adverse responses triggered by Cd-induced stress can be alleviated by the application of LRE + Mt. This intervention bolstered chlorophyll levels and the net photosynthesis rate in plants, by reducing chlorophyll degradation, mitigating excess excitation energy in PSII, and protecting PSII components, particularly D1 protein [71, 76].

Plants of *V. faba* subjected to Cd stress exhibited varied levels of Cd accumulation in their various parts (Table 3). Cd has the capacity to form complexes with proteins, cellulose, pectates, and insoluble Cd phosphate within the root cell wall. Consequently, the accumulation of Cd in the roots impeded the uptake and translocation of other essential nutrients, exacerbating Cd toxicity to the plants. These findings align with prior research [77–79]. It is noteworthy that the amounts of Cd accumulated in the leaves and seeds of *V. faba* grown in contaminated soil surpassed the permissible range for normal plants [80]. Although Cd levels in seeds were lower than those found in other leafy vegetables, including common mallow and cabbage [81].

However, the addition of LRE + Mt as biostimulants can act as chelators by forming complexes with Cd, reducing its mobility and facilitating sequestration in vacuoles or cell walls, ultimately reducing Cd content in plant parts [82]. Licorice root extract contains various bioactive compounds, including glycyrrhizin and flavonoids, which may modify root membrane properties or form complexes with Cd in the rhizosphere, reducing its bioavailability and uptake, simultaneously promoted greater biomass, improved plant nutrition, and provided protecting against abiotic stress [83, 84]. Melatonin has been shown to regulate metal transporters in plant roots, potentially decreasing the influx of Cd into the plant [85].

Cd and salinity induced-stress conditions pronouncedly affected *V. faba* plants. It notably boosted the amounts of total soluble sugars, soluble protein content, proline, phenolic compounds in the leaves, and enhanced DPPH-radical scavenging activity (Table 4). In response to Cd and salinity-induced stress, plants employ sophisticated defense mechanisms encompassing non-enzymatic radical scavengers such as flavonols, flavonoids, and phenolic compounds, to prevent oxidative damage and maintain controlled levels of ROS [86]. These phenolic compounds serve multiple functions, acting as chelators for transition metal ions, scavengers of ROS, and inhibitors of lipid peroxidation by disrupting the radical chain reactions involved in this process. Cd stress induces the production of flavonoids and flavonols, which play a pivotal role in plant protection. These compounds effectively counteract the ROS over-produced by Cd stress by inhibiting the lipoxygenase enzyme responsible for converting polyunsaturated fatty acids into oxygen-containing derivatives. Accumulation of osmotic protectants in leaves could be attributed to reduced water absorption and transport to the shoot under Cd-induced stress. The altered membrane permeability induced by Cd stress seems to create water stress like-conditions, leading to higher proline levels. These findings suggest that plants exposed to Cd stress encountered more damage, necessitating increased soluble sugars and proline to maintain cellular osmotic balance. Cd exposure induces the expression of dehydration related genes and disrupts carbohydrate metabolism, resulting in sugar accumulation. Similar findings have been reported in various studies [30, 87–89]. Furthermore, supplementing with LRE + Mt significantly bolstered defense mechanisms against ROS. Our findings align with those of [28, 90].

Cd stress triggers the production of a substantial quantity of ROS, which can severely impair plant physiology and metabolic processes. To ameliorate the influences of Cd stress, plants activate their antioxidant defense system involving non-enzymatic antioxidants (AsA and GSH) to regulate cellular redox balance and promote the production of phenols, carotenoids, and αToC (Table 5). Enzymatic antioxidants (SOD, CAT, APX and GR) also play a crucial role in neutralizing H_2_O_2_ by converting it into harmless substances (H_2_O and O_2_). GSH plays a pivotal role against ROS-induced oxidative stress damage to cell membranes by preserving zeaxanthins and tocopherols in their reduced forms, which indirectly contribute to membrane protection [67, 91]. Furthermore, GSH prevents denaturation of proteins by inhibiting the oxidation of protein thiol groups under Cd-induced stress. Under oxidative stress, enzymes are triggered, alongside an increase in osmoprotectants and non-enzymatic antioxidants [24, 28]. Interestingly, supplementation with LRE + Mt elevated the concentrations of osmoprotectants and non-enzymatic antioxidants under Cd stress. This supplemental approach seems to reinforce antioxidant defense and osmoregulation mechanisms against Cd stress by modifying cell osmosis, stabilizing membrane structures, and reducing oxidative stress damage [30, 91–96].

In this research, *V. faba* plants subjected to Cd and salinity-induced stress revealed a decline in various growth attributes. However, supplementation with LRE + Mt led to significant improvements (Table 8). The exposure to Cd negatively impacted root growth by inhibiting the mitotic division of meristematic cells [97], and hindering the development of lateral roots [98], leading to necrosis, decomposition, and mucilaginous [25]. This sequence of events in turn disturbs the uptake of vital nutrients and water required for protein synthesis, photosynthesis, and respiration [75, 99, 100], consequently affecting plant growth [101, 102]. Additionally, Cd stress triggered oxidative stress by generating ROS, resulting in damage to cell viability and membranes, and suppressing vital antioxidative enzymes and functional groups necessary for normal plant functions [103]. Conversely, the supplementation with LRE + Mt significantly boosted the growth attributes of *V. faba* plants cultivated in Cd-contaminated saline soil. These findings are consistent with numerous earlier studies [20, 81, 104, 105], which have consistently reported beneficial effects from antioxidants. These substances effectively counteracted the detrimental impacts of Cd and boosted overall plant growth, through increased fresh and dry root and shoot masses, increased stem diameter, larger leaf area, improved nutrient contents, and enhanced processes such as photosynthesis, respiration, cell membrane permeability, nutrients absorption, and hormonal balance.

Cd-induced stress harmed the yield components of *V. faba* plants (Table 9). In response to Cd stress, there was a significant decrease in green pods yield, pods no plant^− 1^, 100 seed weight was recorded, consequently resulting in a marked reduction in the seed yield. This pattern aligns with the findings by [106], who observed that nutrient (Ca, Mn, Cu, and Zn) uptake was inhibited under Cd stress, thereby impeding their absorption and transport to the fruits in paddy rice [107]. Cd stress extend to various biochemical and morphological parameters, including reduced nutrient and water uptake, decreased cell division, disruptions in nutrient uptake and translocation, and heightened oxidative stress. This oxidative stress negatively affects chlorophyll and mitochondria, as well as lipids and proteins, resulting in reduced photosynthesis activity. Consequently, these factors collectively contribute to diminished growth, decreased biomass, and lowered plant yield [23]. However, supplementation with LRE + Mt was found to improve seed yield and quality even under Cd and salinity stress. This supplementary approach alleviated the harmful influences of Cd stress, and significantly promoted yield-related traits [20, 72].

## Conclusions and future prospects

Globally, Cd has emerged as a significant concern in the realm of food safety. This toxic element has detrimental effects on the crop growth and their ability to undergo photosynthesis, ultimately reducing yields and quality. However, the integrative application of licorice root extract and melatonin significantly reduced the adverse effects of Cd on growth, photosynthesis, and enzyme activity. Exogenous licorice root extract and melatonin applied sequentially considerably reduced Cd’s oxidative effects, indicated by decreased oxidative damage. Furthermore, LRE + Mt application induced osmolyte accumulation, activated the antioxidant system, and increased phenol accumulation to counteract the detrimental effects of Cd. Application of LRE + Mt_100_ enhanced Cd tolerance of *V. faba* through improving the physiological status, growth parameters, decreasing Cd levels in leaves and seeds (71%), increasing the activity of antioxidant system, ultimately increasing green pods yield (183%), and seed yield (68%). Although this study could provide a comprehensive understanding of how LRE + Mt affects *V. faba* plants grown in Cd-contaminated saline soil, the molecular mechanisms are unknown. Thus, more research on the mechanisms of LRE + Mt can be worthwhile to unravel the exact mechanisms involved.

## Electronic Supplementary Material

Below is the link to the electronic supplementary material.

Supplementary Material 1

## Data Availability

All datasets generated for this study are included in the article/Supplementary Materials.
