# Supplementary material for: Integrative application of licorice root extract and melatonin improves faba bean growth and production in Cd-contaminated saline soil
Source: BMC Plant Biol. 2025 Jan 8;25:26. doi: 10.1186/s12870-024-05954-0 (PMC11708068; doi:10.1186/s12870-024-05954-0)
Supplement: Supplementary file 1 — Supplementary Material 1 [file 12870_2024_5954_MOESM1_ESM.docx]

Table S1. Chemical analysis of the d licorice root extract (LRE) (on a dry weight basis).

| **Component** | **Unit** | **Value** |
| --- | --- | --- |
| 1. Antioxidants and osmoprotectants: | | |
| Total free amino acids | g kg^−1^ DW | 172 |
| Free proline |  | 36 |
| Soluble sugars |  | 148 |
| Salicylic acid | mg kg^−1^ DW | 29.5 |
| α-Tocopherol |  | 38.4 |
| Glutathione (GSH) |  | 30.2 |
| Ascorbic acid (AsA; Vit. C) |  | 41 |
| Vitamin A (β-carotene) |  | 154 |
| Total B-group vitamins |  | 170 |
| Vitamin E (Tocopherol acetate) |  | 65 |
| Selenium (Se) |  | 0.9 |
| DPPH-radical scavenging activity % |  | 84.6 |
| 2. Phytohormones: | | |
| Total auxins | mg kg^−1^ DW | 4.2 |
| Total gibberellins |  | 5.2 |
| Zeatin-type cytokinin |  | 4.1 |
| 3. Mineral nutrients: | | |
| Nitrogen (N) | mg kg^−1^ DW | 20.2 |
| Phosphorus (P) |  | 21.3 |
| Potassium (K) |  | 47.2 |
| Calcium (Ca) |  | 2.2 |
| Magnesium (Mg) |  | 3.8 |
| Sulfur (S) |  | 2.4 |
| Iron (Fe) |  | 0.94 |
| Manganese (Mn) |  | 0.62 |
| Zinc (Zn) |  | 0.21 |
| Cupper (Cu) |  | 0.02 |
